# Exploration of an instrument measuring outcomes most important to patients through analysis of Overture data

**DOI:** 10.1002/alz70859_105986

**Published:** 2025-12-26

**Authors:** Lily Lee, Evan Hempel, Julia M Leach, Ralph Kern

**Affiliations:** ^1^ Cognito Therapeutics, Cambridge, MA USA

## Abstract

**Background:**

The OVERTURE (NCT03556280) randomized controlled trial (RCT) evaluated Spectris^TM^ in mild‐moderate Alzheimer’s disease (AD)^1^, and collected functional and cognitive outcomes using several validated, clinically relevant instruments. Spectris was associated with significant improvements in ADCS‐ADL and MMSE relative to sham treatment. Linking validated clinical trial measures with outcomes most important to patients and their caregivers is crucial in understanding how well therapies may address patient and family treatment goals. Recent, published studies revealed 42‐concepts that matter most to patients and caregivers in their management of AD (What Matters Most [WMM]).^2^ This research approach revealed specific symptoms, impacts and outcomes most important to patients and caregivers. For example, the desire to maintain independence (eg. taking medications correctly), physical and mental health (eg. not feeling down or depressed), and safety were identified as extremely important.^2^ The 42‐items were mapped to individual queries within assessments commonly used in AD clinical trials.^2^ Herein, we report the results of active and sham treated participants on WMM concepts mapped to instruments used in the Overture study.

**Method:**

Individual items in the ADCS‐ADL, MMSE, ADAS‐Cog, CDR and NPI collected in the Phase 2 Overture study were mapped to each of the 42‐WMM concepts (except #7), adapted from previously described studies.^3^ Mean changes from baseline at Month 6 of each WMM were compared between active and sham groups using T‐Test. An overall score encompassing all improvement measures was also compared (active vs sham) by T‐Test.

**Result:**

Of the 42 WMM concepts described in the literature, 41 were evaluable using instruments used in the Overture study. Twelve of 41 individual WMM concepts (29%) evaluated demonstrated a statistically significant difference (p < 0.05) in favor of active treatment. An exploratory evaluation of mean change from baseline summarizing improvements of the 41 individual components was ‐0.59 for active compared to ‐15.12 for sham treated participants (p=0.0006).

**Conclusion:**

Consideration of outcomes important to patients and their caregivers are important in assessing overall efficacy of AD therapies. Initial analysis of 41 WMM components explored the feasibility of developing a WMM scale demonstrated through application of Overture data, a step towards developing patient‐centric scales.

